# Prevalence and associated factors of psychological distress in tuberculosis patients in Northeast China: a cross-sectional study

**DOI:** 10.1186/s12879-021-06284-4

**Published:** 2021-06-12

**Authors:** Xu Chen, Ruiheng Wu, Jia Xu, Jiawei Wang, Mingcheng Gao, Yunting Chen, Yuanping Pan, Haoqiang Ji, Yuxin Duan, Meng Sun, Liang Du, Ling Zhou

**Affiliations:** School of Public Health, Dalian Medical University, 9 Western Section, Lvshun South Street, Lvshunkou District, Dalian, 116044 Liaoning China

**Keywords:** Tuberculosis patients, Psychological distress, Associated factors, China

## Abstract

**Background:**

Psychological distress, a major comorbidities of tuberculosis (TB) patients, has posed a serious threat to the progress being made in global TB programs by affecting treatment adherence and health outcomes. However, the magnitude and associated factors of psychological distress have not been fully studied in China. The aim of the current study was to assess the prevalence of psychological distress in TB patients and to further determine the effects of socio-demographic characteristics, health-related variables, substance use status, social support, and experienced stigma on psychological distress.

**Methods:**

A cross-sectional survey was conducted among TB patients attending three medical institutions in Dalian, Liaoning Province, Northeast China from November 2020 to March 2021. A structured questionnaire was developed to collect data on patients’ socio-demographic characteristics, health-related information, substance use status, psychological distress, family function, doctor-patient relationship, policy support, experienced stigma and so on. The binary logistics regression model was used to determine the associated factors of psychological distress.

**Results:**

A total of 473 TB patients were enrolled in this study, and the prevalence of psychological distress was 64.1%. Binary logistic regression analysis revealed that patients with a middle school education level or above (*OR*: 0.521, 95%*CI*: 0.279–0.974), no adverse drug reactions (*OR*: 0.476, 95%*CI*: 0.268–0.846), and regular physical exercise (*OR*: 0.528, 95%*CI*: 0.281–0.993) were more likely to stay away from psychological distress. However, patients who had a high economic burden (*OR*: 1.697, 95%*CI*: 1.014–2.840), diabetes (*OR*: 2.165, 95%*CI*: 1.025–4.573), self-rated illness severe (*OR*: 3.169, 95%*CI*: 1.081–9.285), perceived poor resistance (*OR*: 2.065, 95%*CI*: 1.118–3.815), severe family dysfunction (*OR*: 4.001, 95%*CI*: 1.158–13.823), perceived need for strengthen psychological counseling (*OR*: 4.837, 95%*CI*: 2.833–8.258), and a high experienced stigma (*OR*: 3.253, 95%*CI*: 1.966–5.384) tended to have a psychological distress.

**Conclusions:**

The study found that the proportion of psychological distress among TB patients was high in Northeast China, and it was influenced by a variety of factors. Effective interventions to reduce psychological distress in TB patients urgently need to be developed, and greater attention should be given to patients with risk factors.

**Supplementary Information:**

The online version contains supplementary material available at 10.1186/s12879-021-06284-4.

## Background

Tuberculosis (TB) is an ancient chronic infectious disease caused by *Mycobacterium tuberculosis* (MTB), and it mainly occurs in the lungs, with cough, low fever and fatigue as the common symptoms [1]. TB remains a major global public health challenge, as it has a significant impact on morbidity and mortality each year [2]. In 2019, an estimated 10 million people were infected with TB and 1.41 million died from the disease in the world, roughly the same as in 2018 [3]. As a result of COVID-19, there has been a significant drop in the number of officially reported TB cases per month in some high-burden countries between January and June 2020 [3]. The COVID-19 pandemic threatens to reverse the progress made in previous years in reducing the global burden of TB. Models have indicated that the number of people with TB could increase by more than 1 million per year during the 2020–2025 period, and the number of annual TB deaths could rise to the level of 2015 or even 2012 in 2020 and beyond [3]. China has one of the highest burdens of TB in the world. According to the World Health Organization (WHO), the number of new TB cases was about 833,000 in China in 2019, accounting for 8.4% of the global total and ranking third [3]. TB patients often undergo long-term treatment for at least six months and suffer side effects from anti-TB drugs, which can have a serious impact not only on their physical health, but also on their psychological health [4–6].

TB is inextricably linked to psychological health problems, but psychological health problems generally do not receive adequate attention in national TB programs [7, 8]. Studies have indicated that the magnitude of psychological distress in TB patients is high compared to outpatients (excluding TB patients) and the general population, and it is the main comorbidities [9–11]. Psychological distress is a general term that is defined as a psychological state of emotional distress characterized by depression and anxiety [12]. Poverty, chronic illness, unemployment, the loss of a loved one, and many other things can contribute to psychological distress [13]. Psychological distress is very common in comorbidities with other diseases and has become one of the major factors in the global burden of disease [14, 15]. The results of epidemiological studies shown the occurrence of TB and psychological distress as a result of the common risk factors such as poverty and stress [16, 17]. In addition, some TB drugs themselves, such as isoniazid and cycloserine, can also cause adverse psychological reactions [16, 18]. Several studies in Nigeria, Ethiopia, and South Africa reported that the prevalence of psychological distress ranged from 51.9 to 81% in TB patients [13, 19, 20]. A study conducted in three cities in Shandong Province, Eastern China, found that a total of 58.6% of TB patients had moderate and serious psychological distress [21]. Another study, carried out in rural Shandong Province, revealed that 65.2% of TB patients were categorized as having psychological distress [22].

The existence of psychological distress will directly affect the non-adherence treatment of TB patients [23]. A meta-analysis study suggested that patients with depression could have a three-fold higher risk of being non-adherent to treatment than those without depression [24]. More importantly, previous studies have also elucidated that adherence to medication is significantly improved in TB patients who have received multiple psychological sessions [7, 25]. However, non-adherence to treatment can lead to adverse treatment outcomes, such as drug resistance, treatment failure, and even death [23, 26]. It also contributes to a greater economic burden, longer hospital stays and treatment times, increased morbidity, mortality and community transmission [27, 28]. Furthermore, psychological distress may also interfere with an individual’s immune response system, which could result in a low resistance to infection and a poor quality of life [10, 29]. Psychological distress threatens the progress being made in global TB programs and imposes enormous costs on healthcare systems [29]. However, early detection and appropriate psychological interventions can alleviate the psychological distress and its negative consequences in TB patients [7, 30]. Economic modeling also suggests that investing in the prevention, services and management of depression or anxiety in TB patients can yield a high economic return [27]. Therefore, it is important to assess the prevalence of psychological distress among TB patients and its influencing factors if the WHO goal of eliminating TB by 2035 is to be achieved.

Studies conducted in several countries have examined factors that influence psychological distress in TB patients. They determined that psychological distress was more common in TB patients who were female, rural, unmarried, older, less educated, low economic status, co-infected with human immunodeficiency virus (HIV), relapse, multi-drug resistant tuberculosis (MDR-TB), smoking, alcohol use disorders, having at least one other chronic disease, and experiencing TB-associated stigma [10, 11, 13, 20, 25].

In order to design targeted and effective interventions, the prevalence and risk factors of psychological distress in TB patients need to be clearly understood. To our knowledge, no relevant studies have been carried out in Northeast China, and it is not clear whether health status and social support influence psychological distress. However, the prevalence of psychological distress was influenced by geographical location [10, 31]. Therefore, we conducted an epidemiological study in Dalian, Liaoning Province, Northeast China. The aim was to assess the prevalence of psychological distress among TB patients and to further analyze the impact of socio-demographic characteristics, health-related variables, substance use status, social support, and experienced stigma on psychological distress.

## Methods

### Study design and setting

A cross-sectional survey was conducted between November 2020 and March 2021 at three medical institutions in Dalian, Liaoning Province, Northeast China. The three institutions were selected based on the number of patients attending, type of patient and location. They were the Dalian TB hospital, the Zhuanghe TB dispensaries and the Lushun TB dispensaries, respectively. Dalian TB hospital was the only specialized hospital for TB prevention and control in Dalian, and it has complete detection means and advanced medical equipment, in order to adapt to the development of medical undertakings and the needs of patients. Dalian TB hospital was divided into north and south parts, which were located in the convenient transportation of Ganjingzi district and Pulandian district, respectively, and it was the main medical institution for TB patients in Dalian and the referral institution for critically ill patients. Zhuanghe and Lushun TB dispensaries were located in Zhuanghe city (a county-level city) and Lushunkou district, respectively, and only provide treatment for local patients with milder TB. Since January 1, 2011, the Dalian municipal government has issued a policy to benefit the people, providing subsidies for transportation and nutrition expenses to newly registered TB patients (excluding TB pleurisy and other extrapulmonary TB). A total of 245 yuan was given to patients each month. The newly diagnosed patients were given 6 months, and relapse patients were given 8 months.

### Participants

Participants of this study were TB patients who met the inclusion criteria and were treated in Dalian TB Hospital, Zhuanghe and Lushun TB dispensaries between November 2020 and March 2021. The inclusion criteria for patients included the following: (1) TB patients who have been diagnosed in accordance with national TB program guidelines; (2) patients aged 18 years or above; (3) patients without psychosis or communication problems; (4) patients who have no difficulty in understanding the contents of the questionnaire; (5) patients who can comply with the study procedures and agree to participate in the study. In addition, patients who had completed treatment were excluded from the study. A total of 481 TB patients were recruited and completed a structured questionnaire. Of these, 8 questionnaires were deleted due to logical errors or a large number of missing data. As a result, data from 473 patients were eventually included in the study, with a participation rate of 98.3%.

### Data collection

A structured questionnaire was developed to collect data by reviewing a large number of relevant research literature and consulting experts in related fields. Prior to actual data collection, we conducted a pre-survey at the research institution. According to the feedback, the questionnaire was modified and supplemented to ensure its effectiveness. The questionnaire consisted of socio-demographic characteristics, health-related variables, substance use status, psychological distress, family function, doctor-patient relationship, policy support, experienced stigma and so on (See Additional file 1). The data were collected by a fixed team of students from the School of Public Health, Dalian Medical University. Each member of the team received unified training on how to collect data and correct practices to ensure consistent, standardized interviews and data collection.

Socio-demographic characteristics involved gender, age, marital status, educational status, current employment status, residence, immigration and economic burden of TB. The economic burden was categorized as low and high and was decided by the question, “How do you feel about the economic burden of TB on you?”. Health-related variables included TB treatment categories, hospitalization experience, treatment duration, cough, diabetes, the number of currently taking anti-TB drugs, experience of adverse drug reactions, self-rated illness severity, self-perceived body resistance, body mass index (BMI) and physical exercise. Hospitalization experience was assessed by asking “Have you ever been hospitalized for TB?”. Treatment duration was obtained by asking “how long have you been treating TB”. Self-rated illness severity and self-perceived body resistance were collected by asking “Do you think your condition is severe?” and “Do you think you are more likely to get sick than others?”, respectively. BMI was calculated by two questions, “What is your height?” and “What is your weight?”. Physical exercise was determined by the question, “Do you often take part in physical exercise?”. Substance use status referred to the current use of alcohol and cigarettes, and was measured by the questions, “Are you drinking alcohol now?” and “Are you smoking cigarette now?”

Psychological distress was assessed using the Kessler Psychological Distress Scale (K-10) [32]. The scale was a 10-item self-report tool used to measure nonspecific symptoms of depression and anxiety [33]. The participants’ answers to each item were rated on a Likert scale ranging from 1 (none of the time) to 5 (all the time). The total score was the sum of each item score, on a range of 10 to 50. The higher the score, the higher the degree of psychological distress was. Those with scores greater than or equal to 16 were considered to have psychological distress [32]. This scale has been verified in many countries including China [13, 21]. In this study, Cronbach’s a was 0.929.

Family function was measured using the Family APGAR (adaptability, partnership, growth, affection, and resolve) Questionnaire developed and designed by Smilkstein in 1978 [34]. The aim was to assess patients’ satisfaction with social support received from family members based on five components: adaptation, partnership, growth, affection and resolve. Each item in the questionnaire was scored using a 3-point Likert scale ranging from 0 (hardly ever) to 2 (almost always). The item scores were added to give the total score, which ranged from 0 to 10. Higher scores indicated good family function. Family function was divided into three levels, 0 to 3 on a scale of a severely dysfunctional family, 4 to 6 on a scale of a moderately dysfunctional family, and 7 to 10 on a scale of a highly functional family [34]. Family APGAR Questionnaire has been widely used in China and has good reliability and validity [8]. In this study, it has a high internal consistency (Cronbach’s α = 0.937).

In this study, the doctor-patient relationship questionnaire was used to evaluate the relationship between TB patients and doctors [35]. The questionnaire contained eight questions, such as “Are care providers greet you well?” and “Are they giving you adequate contact time?”. The patients were asked to give answers based on their actual feelings during the treatment. Each question had three answers: 1 (never), 2 (sometimes), and 3 (always). The total score ranged from 8 to 24, with a high score reflecting a good doctor-patient relationship. Participants who scored average or above were labeled as having a good doctor-patient relationship [35]. In this study, Cronbach’s α was 0.881.

Three questions were used to measure participants’ satisfaction with national policy support, including their satisfaction with the national free TB policy, the distribution of TB subsidy fees and the medical quality in the hospitals attended. Patients were asked to rate each of the three questions on a scale of 1 (strongly dissatisfied) to 5 (strongly satisfied). The total score ranged from 3 to 15, and patients whose total score was greater than or equal to the average were considered to have a higher degree of satisfaction. The three items had a good internal consistency in our study (Cronbach’s α = 0.838).

Experienced stigma was measured using a 9-item stigma questionnaire developed in accordance with Chinese social and cultural contexts [22]. Items were rated on a 4-point Likert scale, ranging from strongly disagree (=1) to strongly agree (=4). Items were added up to an overall score, which ranged from 9 to 36. The median of the overall score was used as the cut-off value to divide the patients into high and low (including patients who had not experienced stigma) stigma groups [22]. This scale has good reliability and validity. In the current study, its Cronbach’s α was 0.946.

### Data processing and analysis

The integrity and consistency of the data were checked on the time of the receipt of the questionnaires, and the qualified questionnaires were coded. In order to ensure the accuracy of data, double-entry was used to input the data into the database established by EpiData 3.1 (EpiData Association, Odense, Denmark) software. The data was exported to SPSS 21.0 (IBM Corporation, Armonk, State of New York) for statistical analysis. Mean and standard deviation (SD) of continuous data were calculated, and classified data were expressed as frequency and percentage. Chi-square test was used to compare the prevalence of psychological distress in TB patients among different groups. Statistically significant variables in univariate analysis were included in the binary logistics regression model to assess the independent impact of each variable after adjusting for potential confounders. Before the binary logistic regression, we tested the collinearity between the predictive variables. It was found that the variance inflation factor (VIF) of each variable was less than 10, and the tolerance was much higher than 0.1. Therefore, there was no collinearity among all the predictive variables. All the comparisons were two-sided, and *P* < 0.05 was considered statistically significant.

## Results

### Socio-demographic characteristics and their relationship with psychological distress

The 473 TB patients were included in the study, with a mean age of 48.36 years (SD = 17.58). Male participants (69.1%) were more than twice as many as female participants (30.9%). More than half of the participants (65.3%) were married, and 10.1% were divorced or widowed. Almost one-third of the participants (31.9%) had a primary school education or below, and 39.5% of the participants said they were currently unemployed. A large proportion of participants (66.8%) were from rural areas, and a small proportion (20.5%) were migrants. Nearly half of the participants (45.7%) reported a high economic burden from TB. Among the respondents, 303 (64.1%) had psychological distress, and the mean score of psychological distress was 19.62 (SD = 7.49). Univariate analysis indicated that age, educational status, current employment status and economic burden were significantly associated with psychological distress (*P* < 0.05) (Table 1).

**Table 1:** Prevalence of psychological distress according to socio-demographic characteristics of TB patients

Variable	Total n (%)	Psychological Distress n (%)		*P*
		No	Yes	
Gender				
Male	327 (69.1)	121 (37.0)	206 (63.0)	0.471
Female	146 (30.9)	49 (33.6)	97 (66.4)	
Age (years)				
18–30	107 (22.6)	49 (45.8)	58 (54.2)	**0.041**
31–44	82 (17.3)	30 (36.6)	52 (63.4)	
45 or above	284 (60.0)	91 (32.0)	193 (68.0)	
Marital status				
Single	116 (24.5)	44 (37.9)	72 (62.1)	0.138
Married	309 (65.3)	115 (37.2)	194 (62.8)	
Divorced or widowed	48 (10.1)	11 (22.9)	37 (77.1)	
Educational status				
Primary school or below	151 (31.9)	41 (27.2)	110 (72.8)	**0.006**
Middle school or above	322 (68.1)	129 (40.1)	193 (59.9)	
Current employment status				
Employed	286 (60.5)	117 (40.9)	169 (59.1)	**0.005**
Unemployed	187 (39.5)	53 (28.3)	134 (71.7)	
Residence				
Rural	316 (66.8)	104 (32.9)	212 (67.1)	0.051
Urban	157 (33.2)	66 (42.0)	91 (58.0)	
Immigration				
Yes	97 (20.5)	33 (34.0)	64 (66.0)	0.658
No	376 (79.5)	137 (36.4)	239 (63.6)	
Economic burden				
Low	257 (54.3)	122 (47.5)	135 (52.5)	**< 0.001**
High	216 (45.7)	48 (22.2)	168 (77.8)	

### Health-related variables and current substance use status and their relationship with psychological distress

**Fig. 1:**
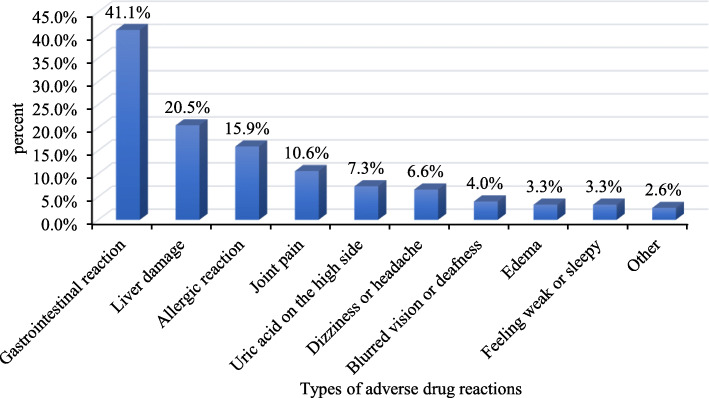
Percentage of types of adverse drug reactions in TB patients

Of the total number of participants, a large number of participants (84.8%) were newly diagnosed patients. Patients who had been hospitalized (79.3%) were nearly four times more than those who had never been hospitalized. The majority of study participants (64.5%) were treated less than 6 months, and nearly one-third of the participants (30.0%) had current cough symptoms. Few of the participants (15.9%) had diabetes. More than half of the participants (51.8%) were currently taking more than 3 anti-TB drugs. In this group, 151 (31.9%) patients reported an adverse drug reaction while taking the drug. Among them, the largest population group (41.4%) was a gastrointestinal reaction (including nausea, vomiting, anorexia, stomach pain, diarrhea, abdominal discomfort, etc.), followed by liver damage (20.5%), allergic reactions (15.9%) (including skin rash, fever, etc.) and joint pain (10.6%), and less were uric acid on the high side (7.3%), dizziness or headache (6.6%), blurred vision or deafness (4.0%), edema (3.3%) and feeling weak or sleepy (3.3%) (Fig. 1). Among participants, nearly three-quarters (70.4%) rated their current ill condition as mild. Perceiving themselves to have a poor and uncertain resistance was found in 24.9 and 20.7% of TB patients, respectively. Almost one-fifth of the participants (18.0%) were currently underweight, and nearly two-thirds (65.3%) never participated in physical exercise. Concerning the current substance use status of the study participants, current rates of alcohol and cigarette use were 11.4 and 22.2%, respectively. Univariate analysis found that TB hospitalization experience, cough, diabetes, adverse drug reactions, self-rated illness severity, perceived poor resistance, and physical exercise were statistically significant with psychological distress (*P* < 0.05) (Table 2).

**Table 2:** Prevalence of psychological distress according to health-related variables and current substance use status of TB patients

Variable	Total n (%)	Psychological Distress n (%)		*P*
		No	Yes	
Treatment category				
New	401 (84.8)	147 (36.7)	254 (63.3)	0.443
Relapse	72 (15.2)	23 (31.9)	49 (68.1)	
TB hospitalization experience				
Yes	375 (79.3)	123 (32.8)	252 (67.2)	**0.005**
No	98 (20.7)	47 (48.0)	51 (52.0)	
TB treatment duration (months)				
< 6	305 (64.5)	117 (38.4)	188 (61.6)	0.315
6–12	150 (31.7)	48 (32.0)	102 (68.0)	
> 12	18 (3.8)	5 (27.8)	13 (72.2)	
Cough				
Yes	142 (30.0)	40 (28.2)	102 (71.8)	**0.021**
No	331 (70.0)	130 (39.3)	201 (60.7)	
Diabetes				
Yes	75 (15.9)	18 (24.0)	57 (76.0)	**0.019**
No	398 (84.1)	152 (38.2)	246 (61.8)	
Current number of anti-TB medicines				
≤ 3	228 (48.2)	85 (37.3)	143 (62.7)	0.558
> 3	245 (51.8)	85 (34.7)	160 (65.3)	
Adverse drug reactions				
Yes	151 (31.9)	36 (23.8)	115 (76.2)	**< 0.001**
No	322 (68.1)	134 (41.6)	188 (58.4)	
Self-rated illness severity				
Mild	333 (70.4)	141 (42.3)	192 (57.7)	**< 0.001**
Moderate	93 (19.7)	23 (24.7)	70 (75.3)	
Severe	47 (9.9)	6 (12.8)	41 (87.2)	
Perceived poor resistance				
Agree	118 (24.9)	25 (21.2)	93 (78.8)	**< 0.001**
Not sure	98 (20.7)	26 (26.5)	72 (73.5)	
Disagree	257 (54.3)	119 (46.3)	138 (53.7)	
Body mass index (kg/m^2^)				
< 18.5	85 (18.0)	26 (30.6)	59 (69.4)	0.256
≥ 18.5	388 (82.0)	144 (37.1)	244 (62.9)	
Physical exercise				
Often	83 (17.5)	41 (49.4)	42 (50.6)	**0.010**
Sometimes	81 (17.1)	31 (38.3)	50 (61.7)	
Never	309 (65.3)	98 (31.7)	211 (68.3)	
Current alcohol use				
Yes	54 (11.4)	23 (42.6)	31 (57.4)	0.279
No	419 (88.6)	147 (35.1)	272 (64.9)	
Current cigarette use				
Yes	105 (22.2)	45 (42.9)	60 (57.1)	0.094
No	368 (77.8)	125 (34.0)	243 (66.0)	

### Social support and perceived stigma and their relationship with psychological distress

The mean score for family function was 8.61 (SD = 2.66). In all, 395 (83.5%) of the patients included in this study were classified as having high family function, 44 (9.3%) and 34 (7.2%) with moderate and severe family dysfunction, respectively. Regarding the doctor-patient relationship of participants, 352 (74.4%) of them were good. More than half of the participants (66.0%) believed that hospitals should strengthen services related to psychological counseling. Almost half of the participants (46.3%) were satisfied with the policy support they received. The mean score for stigma among enrolled patients was 18.86 (SD = 7.14), and 49.0% of patients experienced a high level of stigma. Univariate analysis results showed that there were significant differences in family function, psychological counseling needs, policy support and experienced stigma among participants within the different psychological distress categories (*P* < 0.05) (Table 3).

**Table 3:** Prevalence of psychological distress according to social support status and experienced stigma level of TB patients

Variable	Total n (%)	Psychological Distress n (%)		*P*
		No	Yes	
Family function				
High function	395 (83.5)	151 (38.2)	244 (61.8)	**0.008**
Moderate dysfunction	44 (9.3)	15 (34.1)	29 (65.9)	
Severe dysfunction	34 (7.2)	4 (11.8)	30 (88.2)	
Doctor-patient relationship				
Good	352 (74.4)	134 (38.1)	218 (61.9)	0.100
Poor	121 (25.6)	36 (29.8)	85 (70.2)	
Strengthen psychological counseling				
Need	312 (66.0)	77 (24.7)	235 (75.3)	**< 0.001**
Not too need	161 (34.0)	93 (57.8)	68 (42.2)	
Policy support				
Satisfaction	219 (46.3)	98 (44.7)	121 (55.3)	**< 0.001**
Not too satisfaction	254 (53.7)	72 (28.3)	182 (71.7)	
Experienced stigma				
Low	241 (51.0)	130 (53.9)	111 (46.1)	**< 0.001**
High	232 (49.0)	40 (17.2)	192 (82.8)	

### Predictors of psychological distress among patients with TB

Binary logistic regression analysis revealed that patients who had a middle school education or above (*OR*: 0.521, 95%*CI*: 0.279–0.974), had no adverse drug reactions (*OR*: 0.476, 95%*CI*: 0.268–0.846), and often participated in physical exercise (*OR*: 0.528, 95%*CI*: 0.281–0.993) were more likely to stay away from psychological distress. However, patients with a high economic burden (*OR*: 1.697, 95%*CI*: 1.014–2.840), diabetes (*OR*: 2.165, 95%*CI*: 1.025–4.573), self-rated illness severe (*OR*: 3.169, 95%*CI*: 1.081–9.285), perceived poor resistance (*OR*: 2.065, 95%*CI*: 1.118–3.815), severe family dysfunction (*OR*: 4.001, 95%*CI*: 1.158–13.823), perceived need for strengthen psychological counseling (*OR*: 4.837, 95%*CI*: 2.833–8.258), and a high experienced stigma (*OR*: 3.253, 95%*CI*: 1.966–5.384) were more likely to have psychological distress (Table 4).

**Table 4:** Binary logistic regression analysis of factors associated with psychological distress among patients with TB

Variable	*OR*	95%*CI*	*P*
Age (years)			
18–30	1		
31–44	1.458	0.686–3.099	0.327
45 or above	0.876	0.433–1.772	0.712
Educational status			
Primary school or below	1		
Middle school or above	0.521	0.279–0.974	**0.041**
Current employment status			
Employed	1		
Unemployed	1.414	0.851–2.347	0.181
Economic burden			
Low	1		
High	1.697	1.014–2.840	**0.044**
TB hospitalization experience			
Yes	1.012	0.567–1.805	0.968
No	1		
Cough			
Yes	0.942	0.539–1.646	0.834
No	1		
Diabetes			
Yes	2.165	1.025–4.573	**0.043**
No	1		
Adverse drug reactions			
Yes	1		
No	0.476	0.268–0.846	**0.011**
Self-rated illness severity			
Mild	1		
Moderate	0.870	0.453–1.670	0.675
Severe	3.169	1.081–9.285	**0.035**
Perceived poor resistance			
Agree	2.065	1.118–3.815	**0.021**
Not sure	3.160	1.638–6.095	**0.001**
Disagree	1		
Physical exercise			
Often	0.528	0.281–0.993	**0.047**
Sometimes	0.806	0.417–1.559	0.522
Never	1		
Family function			
High function	1		
Moderate dysfunction	1.150	0.498–2.654	0.744
Severe dysfunction	4.001	1.158–13.823	**0.028**
Strengthening of psychological counseling			
Need	4.837	2.833–8.258	**< 0.001**
Not too need	1		
Policy support			
Satisfaction	0.730	0.451–1.180	0.199
Not too satisfaction	1		
Experienced stigma			
Low	1		
High	3.253	1.966–5.384	**< 0.001**

## Discussion

The current study showed that the prevalence of psychological distress among TB patients was 64.1%. The prevalence was higher than the study carried out in three cities in Shandong Province, Eastern China (58.6%) [21], but similar to another study conducted in rural TB patients in Shandong Province (65.2%) [22]. Compared to studies in other countries, the prevalence was comparable to that in two studies in Ethiopia (63.3 and 67.6%, respectively) [11, 13] and lower than that in South Africa (81%) [20]. The above variations may be due to differences in design, cultural background, socio-economic, and patient population. Of note here, the COVID-19 pandemic had an impact on the care of TB patients in terms of higher diagnostic delay, fewer hospitalizations, and a greater severity of clinical presentations, which may also have contributed to the prevalence of psychological distress in TB patients [36]. In addition, findings also suggested that the magnitude of psychological distress in TB patients was high in Northeast China. The presence of psychological distress can lead to a range of adverse consequences, including poor treatment outcomes [37], and increased morbidity, mortality, and risk of drug resistance [29]. However, psychological distress can be alleviated through appropriate interventions [9, 38]. Therefore, it is critical to explore effective intervention strategies.

In terms of social demographics, the current study did not find a significant association between gender and psychological distress in TB patients. This was different from the study conducted in Angola, but it was consistent with the study in Ethiopia [13, 39]. It has been reported that older age is associated with psychological distress, and we also found this phenomenon in univariate analysis. This may be because older TB patients are a vulnerable group with high mortality rate, and they have more health problems and more frequent adverse drug reactions [40]. Moreover, this study showed that having a primary school education or below was more likely to have psychological distress. Previous studies have indicated that education is a risk factor for depression [41], and common mental disorders are more common among healthy adults with lower education levels [42]. Patients with lower education levels may lack the correct understanding of TB, and often have doubts about whether TB can be cured and emotional insecurity after diagnosis, which can easily lead to psychological distress. Additionally, patients with lower health-related literacy also had higher levels of psychological distress [25]. The study also found a higher rate of psychological distress among TB patients who perceived the high economic burden of their TB treatment. Patients with high economic burden were more likely to have low income and poor family economic conditions, yet both were predictors of psychological distress [11, 43]. In addition, the higher economic burden will make it difficult for patients to pay higher drug costs and review costs over the long term, which will bring great psychological pressure. More importantly, studies have identified higher rates of unemployment among TB patients than those with without TB [25]. Another study also reported that half of patients did not return to normal work even a year after treatment [5]. Not being able to work normally can lead to their income being blocked, which can further strain the finances of families who are not already wealthy and make them feel like a burden on their families, and thus produce psychological distress. Providing economic support to patients may reduce their level of psychological distress and improve treatment adherence, treatment outcomes, and quality of life [20]. Therefore, psychological interventions and related policy development need to give greater consideration to TB patients with lower education and economic status.

Previous studies have shown that having at least one chronic disease in TB patients is associated with psychological distress [13]. Our study also indicated that TB patients with diabetes had a negative impact on psychological distress. This may be because TB patients with diabetes tend to be treated for longer than those with TB alone, which inevitably increases the psychological burden on patients. Additionally, diabetes was considered to be an important risk factor for TB, and poorly controlled diabetes was independently associated with poor TB treatment outcomes [44, 45]. Therefore, more attention should be paid to TB patients who also have diabetes in the screening and diagnosis of psychological distress. Our study also found that patients who experienced adverse drug reactions were more likely to have psychological distress, which is consistent with other studies [46]. Adverse drug reactions can have a negative impact on health, and it is also a serious problem for TB patients [47]. If the patient does not know the cause of the adverse drug reactions, they may be troubled. Hence, it is important to timely detect and guide the adverse drug reactions of patients. The major adverse drug reactions reported by patients were gastrointestinal reactions, liver damage and allergic reactions. The impact of the severity of TB on depression has been proved [7]. Our study further supported this, finding that self-rated having a more severe ill condition was associated with psychological distress. It is understandable that the more severe the ill, the more worried and depressed the patient will be, which may lead to psychological distress [22]. Furthermore, our results also suggested that TB patients with a poor resistance were more likely to suffer psychological distress than other TB patients. This may be due to the fact that patients with poor resistance are often affected by diseases such as influenza, which has a severe impact on their lives, resulting in psychological distress. Physical exercise has been shown to have positive effects on mental health such as anxiety and depression [48]. In our study, TB patients who exercised regularly were found to be more likely to stay away from psychological distress. Physical exercise can improve metabolism, which can help improve health. However, physical health was significantly associated with psychological distress [49]. Therefore, it is beneficial to encourage patients to take part in physical exercise regularly.

Support from others was considered to be an important factor associated with psychological growth [50]. Research has suggested that social support is a protective factor for psychological distress in TB patients [46]. Lack of social support was the most common source of social stress faced by TB patients [31]. Family function is an important source of social support for TB patients, and its effect on psychological distress has been reported [8, 9]. Our study also found that patients with severe family dysfunction were 4 times more likely to experience psychological distress than patients with good family functioning. Good family function brings more positive emotional experience to patients, which helps to increase patients’ life satisfaction and treatment confidence, thereby reducing the degree of psychological distress. Importantly, the majority of patients presented a need for strengthen psychological counseling, and it was found to be significantly associated with psychological distress. This suggests that there is currently a lack of psychological intervention for TB patients. Patients with psychological problems are not identified, let alone treated effectively [20]. Studies have shown that psychological intervention for TB patients has achieved good results in many aspects [7]. Stigma is widespread in TB patients, and those who experience it are more likely to have psychological distress. Studies from Eastern China and Ethiopia back this up [13, 21]. This may be because stigma will cause patients to avoid contact and communication with others and isolate themselves, which makes their feel inferior and prone to psychological distress [51]. Therefore, effective interventions to reduce stigma are needed. This not only reduces the stigma of TB patients, but also the negative effects of stigma, such as psychological distress.

There are some limitations to the current study and the results should be interpreted with caution. First, our study was a cross-sectional study and could not reflect the causal relationship between the predictors and the outcome variables. Therefore, longitudinal studies are needed. Second, this study was conducted only among TB patients in Dalian. Due to the limitation of different social and cultural backgrounds, it should be cautious to extend the current results to other areas with different conditions until the results can be replicated. Third, the study included only TB patients and did not include healthy people as controls. Hence, results may not capture the extent of psychological distress caused by TB alone. Fourth, HIV status was not assessed and it was found to be associated with psychological distress [20]. Finally, our study did not involve qualitative studies for a more comprehensive assessment. Despite these limitations, our study provided valuable information for further research in this area.

## Conclusion

In conclusion, the prevalence of psychological distress in TB patients was high in Northeast China. Educational status, economic burden, diabetes, adverse drug reactions, self-rated illness severity, perceived poor resistance, physical exercise, family function, psychological counseling needs and experienced stigma were associated with psychological distress. Therefore, awareness of the psychological distress of TB patients should be raised, and effective interventions aimed at alleviating the psychological distress of TB patients should be developed. At the same time, more attention should be given to those patients with risk factors in order to produce better clinical outcomes and improve patients’ quality of life.

## Supplementary Information

**Additional file 1.** Questionnaire on mental health status among tuberculosis patients in Dalian. The questionnaire collected information about socio-demographic characteristics, health-related variables, substance use status, psychological distress, stigma, family function, doctor-patient relationship, policy support and so on.

## Data Availability

The datasets used and/or analysed during the current study are available from the corresponding author on reasonable request.

## Abbreviations

TB: tuberculosis
MTB: *Mycobacterium tuberculosis*
WHO: World Health Organization
HIV: human immunodeficiency virus
MDR-TB: multi-drug resistant tuberculosis
BMI: body mass index
K-10: Kessler Psychological Distress Scale
SES: Self-esteem Scale
SD: standard deviation
VIF: variance inflation factor
*OR*: odds ratio
*CI*: confidence interval
