# Supplementary material for: Prevalence and associated factors of psychological distress in tuberculosis patients in Northeast China: a cross-sectional study
Source: BMC Infect Dis. 2021 Jun 12;21:563. doi: 10.1186/s12879-021-06284-4 (PMC8196916; doi:10.1186/s12879-021-06284-4)
Supplement: Supplementary file 1 — Additional file 1. Questionnaire on mental health status among tuberculosis patients in Dalian. The questionnaire collected information about socio-demographic characteristics, health-related variables, substance use status, psychological distress, stigma, family function, doctor-patient relationship, policy support and so on. [file 12879_2021_6284_MOESM1_ESM.pdf]

Questionnaire number: □□□□

Questionnaire on mental health status among tuberculosis patients in Dalian

Hello! We are part of the research group of Dalian Medical University, and we are conducting a survey on the mental health status of patients with tuberculosis (TB). The purpose of this survey was to evaluate the prevalence of psychological distress and related factors among TB patients in Dalian. The data collected will only be used for scientific research and will not have any adverse impact on you personally. We are asking you for your generous help. We assure you that the content of your answers will be kept strictly confidential. If you do not want to participate in the study, you have full right to refuse at any time. But your honest participation will have a great contribution. So please take a few minutes to answer these questions. Thank you for your cooperation!

Do you wish to participate in the study?

……Yes, I want to participate in the study (please go to the next page)

……No, I don't want to participate

Thank you

Part I: Socio-demographic characteristics

No	Questions	Coding Categories	Code
101	Gender	1. Male 2. Female	
102	Age	_______years old	
103	Marital status	1. Unmarried<br>2. Married<br>3. Divorced or widowed	
104	Educational status	1. Primary school or below<br>2. Middle school or above	
105	Current employment status	1. Employed 2. Unemployed	
106	Current residence	1. Rural 2. Urban	
107	Immigration	1. Yes 2. No	
108	How do you feel about the economic burden of TB on you?	1. Low 2. High	

Part II: Health-related variables and substance use status

No	Questions	Coding Categories	Code
201	Treatment category	1. New 2. Relapse	
202	Have you ever been hospitalized for TB?	1. Yes 2. No	
203	How long have you been treated for TB?	1. <6<br>2. 6-12<br>3. >12	
204	Do you have a cough now?	1. Yes 2. No	
205	Do you have diabetes?	1. Yes 2. No	
206	How many anti-TB drugs do you currently take?	1. ≤3 2. >3	
207	Have you had any adverse reactions during taking the drug?	1. Yes, it is_______<br>2. No	
208	Do you think your condition is severe?	1. Mild<br>2. Moderate<br>3. Severe	
209	Do you think you are more likely to get sick than others?	1. Agree<br>2. Not sure<br>3. Disagree	
210	What is your height?	_______m	
211	What is your weight?	_______kg	
212	Do you often take part in physical exercise?	1. Often<br>2. Sometimes<br>3. Never	
213	Are you drinking alcohol now?	1. Yes 2. No	
214	Are you smoking cigarette now?	1. Yes 2. No	

Part III: Psychological distress

In the past 30 days, how often have you been distressed by the following questions?

1 = None of the time 2 = Very little time 3 = Some the time 4 = Most of the time 5 = All the time

No	Questions	Coding Categories					Code
301	How long do you feel tired for no apparent reason?	1	2	3	4	5	
302	How long do you feel nervous?	1	2	3	4	5	
303	How long did you feel so stressed out that nothing calmed you down?	1	2	3	4	5	
304	How long did you feel hopeless?	1	2	3	4	5	
305	How long do you feel restless or irritable?	1	2	3	4	5	
306	How long do you feel restless or irritable to the point of fidgeting?	1	2	3	4	5	
307	How long do you feel depressed?	1	2	3	4	5	
308	How long do you find it hard to do anything?	1	2	3	4	5	
309	How long have you been so sad that nothing can cheer you up?	1	2	3	4	5	
310	How long have you been feeling worthless?	1	2	3	4	5	

Part IV: Experienced stigma

Do you agree or disagree with the following statement?

1 = Strongly disagree 2 = Disagree 3 = Agree 4 = Strongly agree

No	Questions	Coding Categories				Code
401	The local residents are unwilling to chat or have a talk with TB patients.	1	2	3	4	
402	Many residents keep their distance from TB patients.	1	2	3	4	
403	The neighbors will treat the TB patients differently once they get the news.	1	2	3	4	
404	Many people walk away from facing TB patients.	1	2	3	4	
405	My family members feel that they have lost face after knowing my illness with TB.	1	2	3	4	
406	Local residents will not let their kids come close to TB patients.	1	2	3	4	
407	People will not have dinner together with relatives or friends of TB patients.	1	2	3	4	
408	TB patients are not welcome by their local villagers or community residents.	1	2	3	4	
409	I think it is no good to inform the others of my TB infection.	1	2	3	4	

Part V: Family function

Please choose the option that suits you best according to your actual situation.

0 = Hardly ever   1 = Some of the time   2 = Almost always

No	Questions	Coding Categories			Code
501	During my illness, I am satisfied with the help that I receive from my family.	0	1	2	
502	I am satisfied with the way my family discusses items of common interest and shares problem solving with me.	0	1	2	
503	I found that my family accepted the change in my life as a result of my illness.	0	1	2	
504	I am satisfied with the way my family expresses affection and responds to my feelings such as anger, sorrow, and love.	0	1	2	
505	I am satisfied with the amount of time my family and I spend together.	0	1	2	

Part VI: Doctor-patient relationship

Please choose the option that suits you best according to your actual situation.

1 = Never   2 = Sometimes   3 = Always

No	Questions	Coding Categories			Code
601	Are care providers greet you well?	1	2	3	
602	Do you get counseling and education on the disease and its treatment?	1	2	3	
603	Are they giving you adequate contact time?	1	2	3	
604	Are they treating you equally?	1	2	3	
605	Are they motivate or encourages you in the treatment activities?	1	2	3	
606	Are they kept confidentiality regarding all the information that you shared to providers?	1	2	3	
607	Are they kind and Polite?	1	2	3	
608	Do you get the necessary respect from care providers?	1	2	3	
609	Do you think hospitals should strengthen their psychological counseling services?	1. Need<br>2. Not too need			

Part V II: National policy support

Please choose the option that suits you best according to your actual situation.

1 = Strongly dissatisfied 2 = Dissatisfied 3 = General 4 = Satisfied 5 = Strongly satisfied

**No**	**Questions**	**Coding Categories**					**Code**
701	Are you satisfied with the national policy of free TB treatment?	1	2	3	4	5	
702	Are you satisfied with the distribution of tuberculosis treatment subsidy (transportation, nutrition)?	1	2	3	4	5	
703	Are you satisfied with the medical quality (management, facilities, hygiene, etc.) of the hospital you visit?	1	2	3	4	5	

Thank you again for participating in this survey! I wish you a speedy recovery！

Investigate member:__________(signature) The reviewer:__________(signature)

Survey date:_________________
